# A clinical profile of inpatient admissions to the psychogeriatric unit at Stikland Hospital

**DOI:** 10.4102/sajpsychiatry.v25i0.1344

**Published:** 2019-08-26

**Authors:** Durk P. Aartsma, Engelina Groenewald, Liezl Koen, Felix Potocnik, Dana J. Niehaus

**Affiliations:** 1 Department of Psychiatry, Faculty of Medicine and Health Sciences, Stellenbosch University, Cape Town, South Africa

**Keywords:** Elderly, Old Age Psychiatry, Geriatric, Psychogeriatric, Dementia, Neurocognitive Disorders, Inpatient

## Abstract

**Background:**

Globally, the number of older people is rising. As a consequence of greater longevity, an increased burden on both medical and mental health care is expected. As a first step towards developing strategies to provide quality mental health care for this growing population, practitioners need to have a thorough understanding of the composition and needs of these patients.

**Aim:**

To profile the inpatient population of a psychogeriatric unit in terms of demographics, diagnostic makeup, average length of stay and selected outcomes.

**Setting:**

This study was conducted at the psychogeriatric unit of Stikland Hospital, Western Cape, South Africa.

**Methods:**

Demographic and clinical data were retrospectively collected from patient files, discharge summaries and an admission database over a 3-year period.

**Results:**

A total of 903 patients were referred to Stikland Hospital during a 3-year period. Of the 498 patients who were admitted, 56 were readmissions. The mean age of patients was 67 years, and more than 57% of patients were female. The majority of patients (97.1%) were admitted as involuntary mental health users. The diagnosis of a cognitive disorder was made in 49.5% of admissions followed by psychotic disorders in 36.9% and mood disorders in 23.2%. The median length of stay was 53 days.

**Conclusion:**

The findings of this study illustrate that mental health services for the elderly in the Western Cape are insufficient, as only patients with severe illness and comorbidity could be admitted. The study emphasises the need for the restructuring of resources and the implementation of strategies, which may decrease the frequency of admissions to inpatient geriatric units.

## Introduction

In 2015, 12% of the world’s population was more than 60 years old. It is expected that this number will rise to 22% by 2050.^1^ The ageing of the global population represents a major mental health concern because mental disorders such as neurocognitive disorders, depression, anxiety disorders and substance disorders become more prevalent with increasing age.^2^ Presently, almost 20% of people over the age of 60 suffer from a mental disorder,^2^ and by 2030, this number will double.^3^ Major neurocognitive disorder (dementia) is one of the most prevalent mental disorders in the elderly, and in 2017, 50 million people were living with this disorder globally.^2^ This number is expected to increase to 82 million by 2030 and 152 million by 2050.^2^ Depression is also common in the elderly with prevalence estimates of 7% in the general elderly population,^2^ 6% – 10% in primary care settings and 30% in medical inpatients and long-term care settings.^3^. Mental disorders cause significant impairment in the quality of life of elderly patients, their caregivers and their family members.^2^ These disorders also exacerbate the disability associated with other medical conditions and result in increased utilisation of health services.^2,3^

This demographic transition is not unique to high-income countries.^3,4^ It is expected that 80% of the elderly will live in low- and middle-income countries by 2050,^1^ and the greatest burden of dementia is concentrated in these countries.^2^ Similarly, in South Africa, the number of the population older than the age of 60 increased from 2.8 million (7.1% of the population) in 1996 to 4.1 million (8% of the population) in 2011. It is projected that this number will continue to rise and that approximately 7 million people will be older than 60 in 2030.^5^

The prevalence of psychiatric disorders in South African elderly is nevertheless unknown. The South African Stress and Health (SASH) study estimated the lifetime prevalence of any psychiatric disorder in adults older than 65 years to be 27.9%.^6^ The lifetime prevalence of anxiety disorders was 17%, mood disorders was 6.5%, and substance disorders was 11%. However, the 12-month prevalence of these disorders was not estimated in adults older than 65, and the prevalence of other psychiatric disorders was not investigated in this study. Research on the prevalence of dementia in South Africa is also limited.^7^ The only large community prevalence study to date estimated an 8% prevalence rate for dementia in elderly people living in rural Eastern Cape.^8^ Other studies which quantified the prevalence of dementia in South Africa were either very small^9,10^ or based only on clinical samples.^11,12^

South Africa does not have adequate resources to treat the elderly with mental disorders. South Africa has < 5 old-age psychiatrists and only 1 psychogeriatric training unit to cater for a current population of 57.7 million people.^13,14^

The growing elderly population places an increasingly greater demand on over-burdened and under-resourced inpatient psychogeriatric services. A thorough understanding of the patient population using these services is necessary to inform clinical decision-making, focus service development and to make timely provisions for the predicted needs of the elderly.^15^ The aim of this study was to profile geriatric inpatients at Stikland Hospital. It is expected that an insight into the clinical, socio-demographic and readmission characteristics of this age group will reflect on the service requirements and serve as a first step to improving care and accessibility of treatment for the elderly in South Africa.

## Methods

### Study design

We conducted a retrospective record review of patients admitted to the psychogeriatric inpatient unit at Stikland Hospital between 01 January 2013 and 31 December 2015.

### Study setting

Stikland Hospital is a public, tertiary psychiatric hospital situated in the Western Cape Province, which has a total population of more than 6 million people, including 594 266 persons older than 60.^14^ The province is divided into two catchment areas which are serviced by two tertiary academic hospitals, Groote Schuur and Tygerberg Hospital. Stikland Hospital is the only geriatric psychiatry public sector-funded inpatient unit, and it takes referrals from across the entire province.

The psychogeriatric unit at Stikland Hospital consists of three wards with a total bed capacity of 77 (33 male beds and 44 female beds). Once a referral has been reviewed and the documentation is found to be in order, the patient is added to the waiting list. The required documentation is a referral letter and correctly completed *Mental Health Care Act* (MHCA) forms. As soon as a patient is discharged and a bed becomes available, the first patient on the waiting list is accepted for admission.

### Study population

The records of all patients admitted between 01 January 2013 and 31 December 2015 were included in the study. Patients younger than 60 years of age were excluded from the study.

### Data abstraction

The following information were extracted by the principle investigator and subsequently collated on an anonymised Microsoft Excel spreadsheet:

#### Admission data

Admission status according to the *Mental Health Care Act* 17 of 2002, the name of the referring hospital, the date of referral, the date that all administrative documents were in order, the date that a patient was put on the waiting list, the date of admission and discharge, the number of admissions during the 3-year review period and the age of the patient at the first admission during this study period.

#### Demographic data: Age and gender

**Diagnoses:** Primary psychiatric diagnoses, as well as medical and/or psychiatric comorbidities, were recorded. The first psychiatric diagnosis listed on the discharge summary was considered the primary diagnosis and subsequent diagnoses were recorded as comorbid conditions. Psychiatric disorders were diagnosed according to the Diagnostic and Statistical Manual of Mental Disorders 4th edition, Text Revision (DSM-IV, TR).^16^ The primary psychiatric diagnosis was grouped into one of four diagnostic categories, as summarised in Table 1.

**TABLE 1:** Major categories used for diagnosis of psychiatric disorders according to the Diagnostic and Statistical Manual of Mental Disorders 4th edition, text revision.

Diagnostic category	Disorder type
Mood disorders	Major depressive disorder, bipolar disorder, dysthymic disorder, cyclothymic disorder, mood disorder due to a general medical condition, substance-induced mood disorder
Psychotic disorders	Schizophrenia, Schizoaffective disorder, Psychotic disorder due to a general medical condition, substance-induced psychotic disorder
Cognitive disorders	Dementia of the Alzheimer’s type, vascular dementia, dementia due to a general medical condition, substance-induced persisting dementia, dementia due to multiple aetiologies, dementia not otherwise specified
Other psychiatric conditions	Anxiety disorders, substance-related disorders, adjustment disorders

### Analysis of data

Continuous variables were summarised as mean ± standard deviation (s.d.), or median and standard deviation. Nominal variables were summarised as counts and percentages. Group differences were tested using Student’s *t*-test (or the Mann-Whitney U-tests for non-parametric data) and Chi-square tests. All analyses were performed using SPSS, version 25, and statistically significant differences were established at *p* < 0.05.

## Ethical consideration

The study was approved by the Health Research Ethics Committee of Stellenbosch University (study number: S14/08/163), as well as the Head of Establishment (Stikland Hospital). A waiver of informed consent was granted for this retrospective study. All identifiable patient information was anonymised.

## Results

During the 3-year study period, 903 referrals were placed on the waiting list of which one was discovered later to be 54 years of age. We, therefore, report only on 902 referrals. 491 (54.4%) of the referred patients were admitted between the ages of 60 and 89 (mean age 66.92 years, s.d. 5.93). More females than males were admitted over the period. Eighty-eight (17.9%) of the admitted patients had one to four readmissions during this period, and 54 (6%) patients were still in hospital (*n* = 46) or on the waiting list (*n* = 8) by the end of the study period. The mean waiting time from being added to the waiting list until admission to the unit was 18 days, and females waited longer (22 days, s.d. 17.74) than males (14 days, s.d. 11.61; *p* < 0.0001). The mean time spent waiting for admission increased significantly (*p* < 0.0001) from 12 days in 2013 to 22 days in 2014 and 22 days in 2015.

The number of referrals from the two catchment areas was similar. The Groote Schuur area referred 443 patients (49.1%), of which 238 (52.6%) were admitted, whereas the Tygerberg area referred 459 patients (50.9%) of which 258 (56.2%) were admitted. There were no significant differences in terms of percentage admitted from the waiting list (*p* = 0.285 Chi-sq). Groote Schuur Hospital itself sent the most (23.3%) referral letters (*n* = 210) and also had the highest number of admissions (11.8%, *n* = 106), followed by Karl Bremer Hospital (11.1%), Victoria Hospital (8.9%) and New Somerset Hospital (8.0%) during the study period.

The median length of stay was 53 days (mean 70.71, s.d. 63.1, range 1–552 days) and was not significantly influenced by age (*p* = 0.707) or gender (*p* = 0.946). The primary diagnosis and the presence of comorbid medical and psychiatric disorders were not significantly associated with length of stay.

The number of readmissions over the 3 years varied from one to four admissions, but most patients (88.8%) were admitted only once. Female patients had a slightly higher readmission frequency compared to males.

Almost all patients (97.1%) were admitted as involuntary patients under the *Mental Health Care Act* 17 of 2002.

The most common single major psychiatric disorder diagnoses in both males and females were cognitive disorders (24.8%), followed by psychotic disorders (23.4%) and mood disorders (10.6%) (Table 2). As part of a single or comorbid diagnosis, cognitive disorders remained the most common with 49.5% of cases (*n* = 243), while psychotic disorders were part of the diagnosis in 36.9% (*n* = 181), mood disorders in 23.2% (*n* = 114) and substance-related disorders in 10.8% (*n* = 53). The most frequent psychiatric comorbid diagnoses were substance-related disorders and Vascular Dementia (*n* = 79) and substance-related disorders (*n* = 51). Of the 243 patients with cognitive disorders, the most common types of cognitive disorders were Vascular Dementia (63.4%, *n* = 154), Dementia Not Otherwise Specified (19.8%, *n* = 48) and Dementia Due to Multiple Aetiologies (8.6%, *n* = 21). 5.8% of patients (*n* = 14) had Dementia of the Alzheimer’s Type and 2.5% (*n* = 6) had Dementia Due to a General Medical Condition.

**TABLE 2:** Diagnostic categories and comorbid psychiatric conditions of patients (*n* = 491)†

Characteristic	Overall (*n*)	%
**Diagnostic categories**		
Cognitive disorder only	122	24.8
Cognitive disorder and anxiety disorder	2	0.4
Cognitive disorder and substance-related disorder	18	3.7
Cognitive disorder and mood disorder	46	9.4
Cognitive disorder and mood disorder and substance-related disorder	4	0.8
Cognitive disorder and psychotic disorder	46	9.4
Cognitive disorder and psychotic disorder and substance-related disorder	5	1.0
Psychotic disorder	115	23.4
Psychotic disorders and substance-related disorder	15	3.1
Mood disorder	52	10.6
Mood disorder and substance-related disorder	9	1.8
Mood disorder and anxiety disorder	3	0.6
Substance-related disorder	2	0.4
Eating disorders	1	0.2
Other	51	10.4

† , Patients that were readmitted were counted as separate cases.

There was a high prevalence (74.5%) of medical comorbidities within this population (Table 3). The number of medical comorbidities ranged from 1 to 4 (1 = 27.5%, 2 = 25.3%, 3 = 13.6%, 4 = 7.9%) and did not impact the length of stay (*p* = NS). Hypertension and hypercholesterolemia were the most prevalent medical comorbidities in this sample (Table 3).

**TABLE 3:** Frequency of the most prevalent medical comorbidities.

Variable	Frequency (*n*)	%
**Medical comorbidities**		
Hypertension	208	47.4
Hypercholesterolemia	91	20.7
Diabetes mellitus	64	14.6
Vitamin B12 deficiency	43	9.8
Chronic obstructive pulmonary disease	31	7.1
Hypothyroidism	26	5.9

## Discussion

This 3-year retrospective review of admissions to a psychogeriatric unit in the Western Cape provides a broad overview of patient characteristics and emphasises the array of psychiatric disorders and comorbidities affecting this population.

### Admissions

The Stikland Hospital psychogeriatric unit is the only specialised inpatient unit for elderly patients with psychiatric disorders in the Western Cape. Yet, only approximately half of the patients who required further inpatient care at the unit were admitted during this time period. Of the 411 patients on the waiting list who were not admitted, only 8 remained on the waiting list at the end of the study. The remaining 403 patients were probably discharged from the referring hospitals due to bed pressure, and improvement in their symptoms while they were waiting for admission to Stikland Hospital.

The time that patients had to wait for admission to the unit increased markedly from 2013 to 2015. The actual waiting time is probably much longer than the period demonstrated in this study (22 days in 2015) as patients were only added to the list once the referring agent provided the correct referral documents. Females waited significantly longer than males, which could be explained by the fact that the unit does not have an adequate number of female beds. It is apparent from these findings that the Stikland psychogeriatric unit is unable to meet the increasing demand for psychogeriatric services in the Western Cape Province.

The median length of stay of 53 days is much longer than similar international studies (median 28 and 29 days).^15,17^ Due to the complexity of psychiatric conditions in the elderly, these patients often require a longer hospital admission. Similar international studies demonstrated a strong association between the number of admissions, the length of stay and the psychiatric diagnosis: psychotic and mood disorders required longer and more frequent admissions than other psychiatric conditions.^15,17^ These associations were not demonstrated in our study.

Involuntary care was required in the majority of patients (97.1%). This number is much higher than in psychogeriatric units in developed countries^15^ and the general adult psychiatric population in South Africa (75.6%).^18^ The high number of involuntary admissions to our unit indicates that the unit mostly admitted patients with a severe mental illness, who posed a high risk to themselves and others. Patients who could consent to admission (assisted and voluntary patients) did not get the opportunity to be admitted to the psychogeriatric unit due to the shortage of beds.

### Demographic information

The mean age of patients in this study was 66.92 years. This is much lower than a similar study conducted in Australia^15^ where the mean age of 516 patients admitted to an aged psychiatry facility was 74.7 years. The lower mean age in our study could be explained by the fact that the majority of South African elderly is between 60 and 64 years old, and that South Africa has a much lower median population age than many high-income countries.^5^ Similar to our study, a high proportion of patients in the Australian study were female.^15^ The high number of females in our study could be explained by the high proportion of elderly females in the South African population.^5^

### Diagnosis

There were significant differences between the diagnostic profiles of patients admitted to this unit compared to units in developed countries. The most common diagnosis in our unit was cognitive disorders (49.5%) followed by psychotic disorders (36.9%). In a similar study conducted in the United States,^19^ 62.5% of inpatients had an affective disorder, whereas an Israeli study^20^ reported equal rates of affective disorders (31.6%) and psychosis (32.1%). In the Australian study,^15^ the most common primary diagnosis was affective disorders (39%) followed by dementia (27.5%) and psychotic disorders (25.8%). The differences in the diagnostic profiles of these units are probably not due to a higher prevalence of neurocognitive and psychotic disorders in less-developed countries but rather due to the limited resources available and a shortage of appropriate caring facilities and social support for patients in developing countries.^5,13^ It also emphasises that our unit is only able to admit the most severe cases of behaviourally disturbed and disruptive patients and is therefore unable to provide care for patients with affective disorders that may warrant admission.

There was a high rate of substance misuse in our population: 10.8% of the patients in our study were diagnosed with substance-related disorders. This is higher than in similar international studies^15^ and emphasises that substance misuse is a noteworthy concern in older patients.

The prevalence of medical comorbidities in this study was high (74.5%). People with psychiatric illness generally have higher rates of medical comorbidity compared to the general population for various reasons.

These include both patient factors (such as unhealthy behaviours, poor adherence to treatment and limited help-seeking and attendance of appointments) and healthcare factors (fewer investigations and less availability and quality of medical advice).^21^ This becomes even more pronounced with advanced age.

Elderly patients with mental disorders had more medical comorbidities than those without mental disorders, and these comorbidities are often detected late and inadequately managed.^22^ Common medical comorbidities in this study included hypertension, hypercholesterolemia and diabetes mellitus. Considering the high rates of cardiovascular risk factors of patients in this group, the high number of patients with a vascular dementia is, perhaps, unsurprising.

Multiple patients had a documented Vitamin B12 deficiency (9.8%). A local study found a similar prevalence of Vitamin B12 deficiency in the general elderly population.^23^ Although the aetiology of Vitamin B12 deficiency is multifactorial, the high number of patients with Vitamin B12 deficiency could be explained by the poor nutritional status of elderly people in South Africa.^23^

### Limitations and recommendations

This study involved patient data collected over a period of 3 years, which provides a comprehensive overview of patients admitted to the unit. However, the study was a retrospective record review and therefore depended on the quality of the information in the patients’ folders. Diagnoses were made by the clinical team according to the DSM IV-TR but were not generated with standardised assessment instruments. The treating clinician may have had difficulty distinguishing the primary and comorbid diagnoses, as there is often an overlap in symptomatology and aetiological factors of psychiatric disorders.

## Conclusion

The findings of this study illustrate that the single psychogeriatric inpatient unit in the Western Cape Province is only able to admit high-risk patients with severe mental illness and multiple comorbidities. This sole unit is therefore not sufficient to meet the needs of aged mentally ill patients in the province. The study emphasises the need for the restructuring of resources, further research, and the implementation of other strategies (such as intensive outpatient programmes and other specialised services), which may decrease the frequency of admissions to inpatient geriatric units. The number of these specialised units should also be increased – not only in the Western Cape but also in the rest of the country.
